# Differences in standing and sitting spinopelvic sagittal alignment for patients with posterior lumbar fusion: important considerations for the changes of unfused adjacent segments lordosis

**DOI:** 10.1186/s12891-020-03777-2

**Published:** 2020-11-18

**Authors:** Zhuoran Sun, Siyu Zhou, Wei Wang, Da Zou, Weishi Li

**Affiliations:** 1 Orthopaedic Department of Peking University Third Hospital, No 49. North Garden Street, HaiDian District, Beijing, 100191 China; 2 Beijing Key Laboratory of Spinal Disease Research, Beijing, China; 3 Engineering Research Center of Bone and Joint Precision Medicine, Ministry of Education, Beijing, China

**Keywords:** Sitting, Standing, Sagittal alignment, Unfused adjacent segment lordosis, Lumbar lordosis

## Abstract

**Objective:**

This study aimed to describe the changes in spinopelvic sagittal alignment in the sitting position after posterior lumbar fusion, and to identify the factors influencing unfused adjacent segment lordosis.

**Methods:**

Consecutive patients with lumbar degenerative disease who underwent posterior lumbar interbody fusion between December 2010 and April 2012 were recruited. Lateral full spine radiographs were obtained in the standing, erect sitting, and natural sitting positions. Spinopelvic parameters were measured preoperatively and at the final follow-up.

**Results:**

The data of 63 patients were analyzed in this study. The average age was 61.6 ± 11.0 years. When changing from standing to sitting at the final follow-up, all spinopelvic sagittal parameters with the exceptions of pelvic incidence and thoracic kyphosis were significantly altered. The most noticeable changes occurred in the natural sitting position, with the spine slumped toward achieving a C-shaped sagittal profile. Multiple linear regression analysis revealed that when changing to a natural sitting position, age and fusion levels reflected the changes in lumbar lordosis (ΔLL), age and lumbosacral fusion reflected the changes in upper residual lordosis (ΔURL).

**Conclusion:**

The most noticeable changes in spinopelvic sagittal alignment occurred in the natural sitting position after lumbar fusion. Age, fusion levels, and lumbosacral fusion significantly influenced the differences in LL and URL between the standing and natural sitting position. These characteristics should be fully considered when planning spinal realignment surgery and investigating the etiological factors of junctional complications.

## Background

Due to the changes induced by modern lifestyles, people are spending increasingly more time in the sitting position, with many actually spending more time sitting than standing. It is therefore important to understand the effects of the sitting position on changes in lumbar alignment and pelvic compensation. Previous studies have investigated the basic changes occurring in the lumbar and pelvic regions when moving from the standing to sitting position in healthy subjects, which can be summarized as a straightened curve in the lumbar region (decreased lumbar lordosis) and pelvic retroversion (increased pelvic tilt) [1–5]. Spinal sagittal alignment is reported to greatly affect the clinical outcomes and quality of life [6, 7]. Restoring sagittal balance is an important goal of surgical treatment for patients with lumbar degenerative disease, but the ideal reference values for surgical planning are predominantly based on standing radiograph results [8, 9]. The spinopelvic sagittal alignment and sagittal balance in the sitting position after lumbar fusion have not been well discussed, and the changes in the sagittal alignment of unfused adjacent segments are also unclear.

Therefore, the present study aimed to analyze the changes in spinopelvic sagittal alignment in the sitting position after lumbar fusion, and to determine the factors influencing unfused adjacent segment lordosis in the sitting position.

## Methods

### Patients

This study was approved by the ethics committee (IRB00006761–2018192) and was performed according to the principles of the Declaration of Helsinki. We recruited consecutive patients who underwent posterior lumbar interbody fusion (PLIF) for lumbar degenerative disease at our hospital between December 2010 and April 2012. Written informed consent was obtained from all patients. All patients met the following inclusion criteria: patients who received PLIF with the lower instrumented vertebra located in the lumbar or sacral regions, and fusion levels of ≤4. The exclusion criteria were as follows: patients (1) who underwent other spinal surgeries, (2) with coronal deformity and adjacent segment instability, (3) with severe lower back pain affecting sitting and standing position or an Oswestry Disability Index of > 40, (4) with hip or knee joint contracture, (5) with vertebral fracture, (6) with neuromuscular disorders, (5) with severe osteoporosis, and (7) with internal fixation breakage or pseudarthrosis formation.

### Assessment

All enrolled patients underwent comprehensive history taking and a physical examination. Sex, age, body height, and body mass index (BMI) were recorded. Computed tomography (CT) was then performed to evaluate the fusion, and X-ray imaging of lumbar extension and flexion was performed to assess adjacent segment stability.

**Fig. 1:**
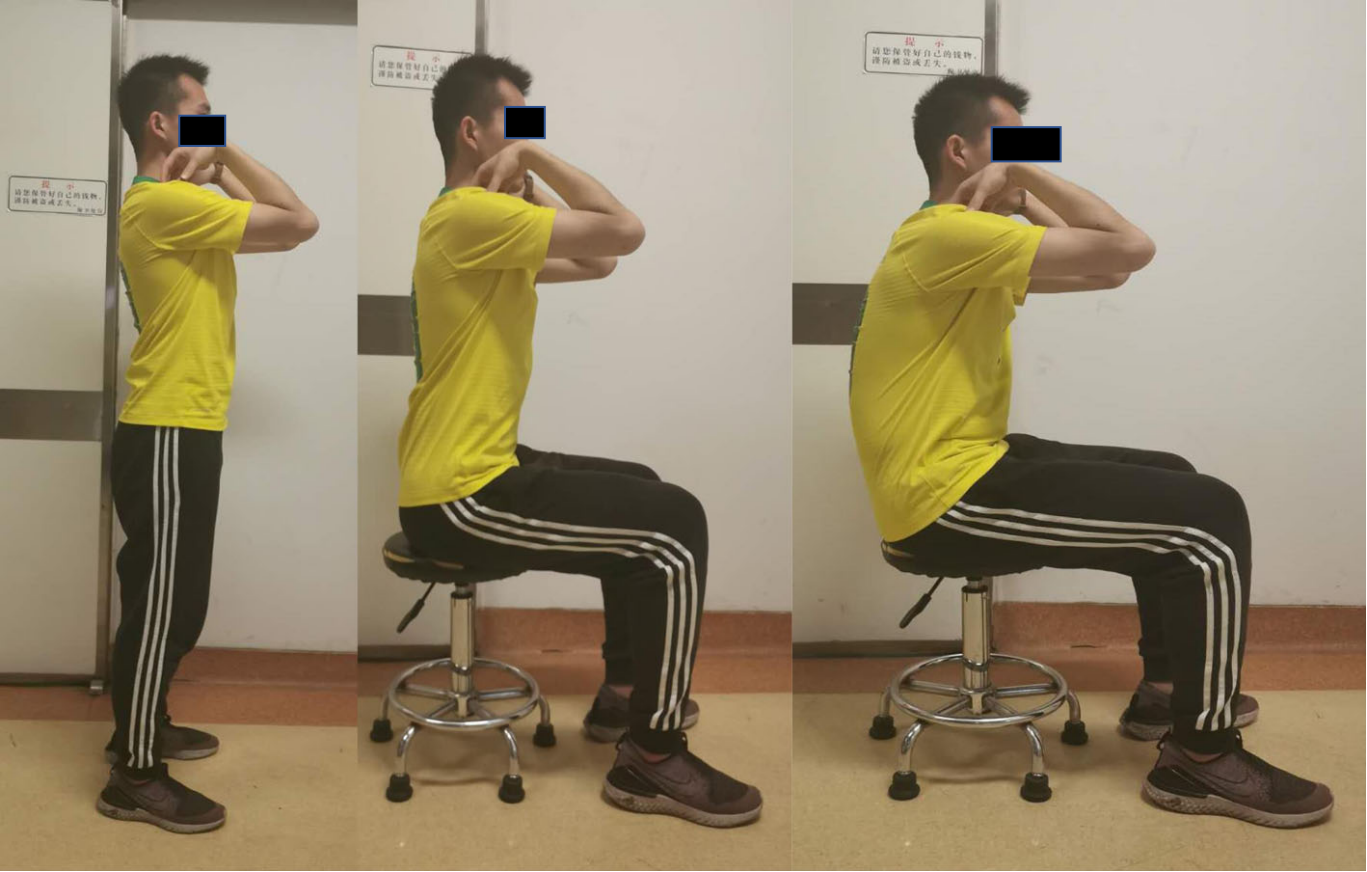
Photographs to instruct the patients in standing (left), erect sitting (middle) and natural sitting positions

For each patient, lateral full standing and sitting radiographs of the spine were obtained with a constant distance between the subject and the radiographic source (Fig. 1). For standing radiographs, the patients were instructed to stand as straight as possible, with the fingers touching the homolateral collar bones [10]. In the erect sitting position, they were asked to flex their hips and knees to 90^°^, and sit as straight as possible, with the fingers touching the homolateral collar bones [11]. In the natural sitting position, the patients were instructed to sit as naturally as they would on a chair while relaxing the trunk. A height-adjustable stool without a back-rest was provided such that the height could be adjusted to achieve a standardized posture, with the feet placed flat on the ground. If the patients’ feet could not touch the ground after adjusting the stool height, a wooden step was provided.

**Fig. 2:**
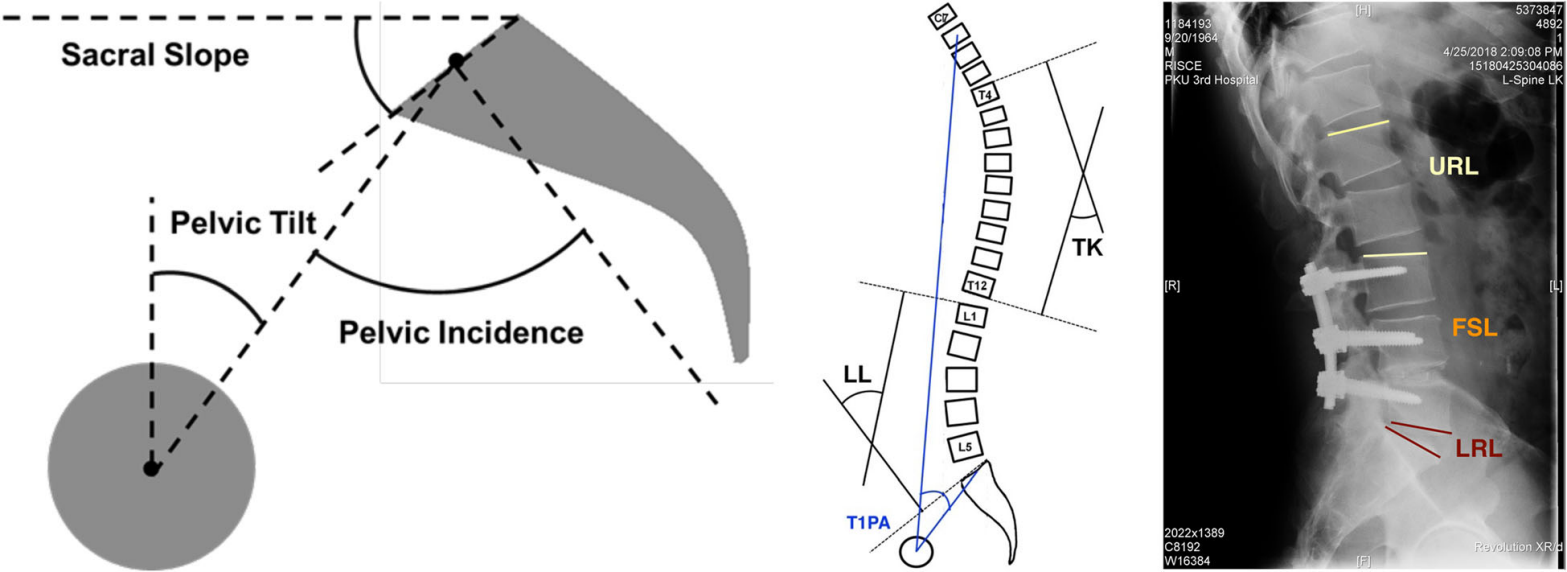
Schematic diagram of measured parameters

The radiographs were digitized, and all measurements were performed by means of imaging software (Centricity RIS/PACS, GE Healthcare), based on standard measurement techniques by two senior spine surgeons, and the average of their results was recorded. The following parameters were measured preoperatively and at the final follow-up (Fig. 2): (1) Pelvic parameters: pelvic incidence (PI), pelvic tilt (PT), and sacral slope (SS); (2) Local curvature: lumbar lordosis (LL), fusion segment lordosis (FSL) i.e. the angle between the upper and lower endplates of the fusion level (endplate of S1 in lumbosacral fusion), upper residual lordosis (URL) i.e. the angle between the upper endplate of L1 and the upper endplate of the fusion level, lower residual lordosis (LRL) i.e. the angle between the lower endplate of the fusion level and the upper endplate of S1, and thoracic kyphosis (TK); (3) Global parameters: T1 pelvic angle (T1PA) i.e. the angle between the line from the femoral head axis to the centroid of T1 and the line from the femoral head axis to the middle of the S1 endplate. The changes in LL, unfused adjacent segment lordosis, and PT between standing and natural sitting were calculated.

### Statistical analysis

All data were analyzed using SPSS software (version 17.0; SPSS, Chicago, IL). Inter-observer reliability was assessed using the intraclass correlation coefficient (ICC). An ICC of ≥0.80 was considered to indicate excellent reliability. Continuous variables are expressed as means ± standard deviation. An adaptation of the Kolmogorov-Smirnov test was applied to assess the normality of the distribution. The changes in parameters were analyzed using a one-way analysis of variance (or the Kruskal-Wallis test) with post hoc comparisons performed among different positions. We set the physical and surgical factors (sex, age, body height, BMI, lumbosacral fusion, fusion levels, and PI) as explanatory variables. Correlations between the changes in lordosis and these factors were analyzed using Spearman correlation analysis. Finally, multiple regression analyses with a forward stepwise procedure were conducted. A *p* value of < 0.05 was considered to indicate a statistically significant difference.

## Results

The data of 63 patients (33 females and 30 males) were analyzed in this study. The average age of the patients was 61.6 ± 11.0 years (range 31–81 years). The mean BMI was 26.6 ± 3.6 kg/m^2^ (18.7–39.6 kg/m^2^). The mean follow-up time was 81.7 ± 7.3 months (range 68–94 months). Thirty-nine patients underwent lumbosacral fusion: L5-S1 fusion in three cases, L4-S1 fusion in 20 cases, L3-S1 fusion in 15 cases, and L2-S1 fusion in one case. Twenty-four patients underwent lumbar floating fusion: L3-L5 fusion in 13 cases, L4-L5 fusion in five cases, and L2-L5 fusion in six cases.

All parameters were measured with good reproducibility. The inter-observer mean ICC was 0.93 (range 0.82–0.99). The mean PT was 14.5 ± 9.0^°^, SS was 31.9 ± 8.1^°^, and LL was 41.7 ± 12.8^°^ in the standing position before surgery, while the FSL was 26.3 ± 9.3^°^ at the final follow-up. The values of these parameters in the standing, erect sitting, and natural sitting positions are provided in Table 1. The PT, SS, and LL values in the standing position did not significantly differ before and after surgery.

**Table 1:** Comparison of spinal-pelvic sagittal parameters in sitting versus standing position

Parameters	Floating fusion group				Lumbosacral fusion group			
	Standing	Erect sitting	Natural sitting	*P* (ANOVA)	Standing	Erect sitting	Natural sitting	*P* (ANOVA)
PI (^°^)	49.4 ± 9.0^a^	50.4 ± 9.3^a^	49.5 ± 8.7^a^	0.905	47.3 ± 9.1^a^	48.3 ± 10.0^a^	49.4 ± 10.7^a^	0.648
PT (^°^)	15.8 ± 6.5^a^	20.4 ± 8.8^a^	25.6 ± 10.5^b^	0.001	16.0 ± 6.0^a^	19.9 ± 10.1^a^	26.2 ± 10.0^b^	< 0.001
SS (^°^)	33.6 ± 9.0^a^	30.0 ± 7.8^a^	23.9 ± 8.1^b^	0.001	31.1 ± 6.7^a^	28.2 ± 9.0^a^	23.0 ± 9.0^b^	< 0.001
LL (^°^)	45.9 ± 10.4^a^	38.5 ± 10.8^b^	31.5 ± 11.9^c^	< 0.001	41.8 ± 10.9^a^	35.1 ± 13.2^b^	29.0 ± 13.1^c^	< 0.001
TK (^°^)	32.7 ± 10.8^a^	31.1 ± 12.7^a^	35.2 ± 11.5^a^	0.468	30.2 ± 10.6^a^	28.4 ± 11.6^a^	33.2 ± 11.1^a^	0.160
URL (^°^)	8.8 ± 6.9^a^	4.6 ± 6.7^a^	1.0 ± 8.0^b^	0.002	13.3 ± 10.7^a^	4.6 ± 10.6^b^	− 0.5 ± 9.1^c^	< 0.001
LRL (^°^)	14.1 ± 5.8^a^	11.7 ± 6.4^a^	8.0 ± 5.4^b^	0.002	–	–	–	
TPA (^°^)	11.4 ± 7.2^a^	18.0 ± 7.8^b^	24.6 ± 9.4^c^	< 0.001	12.4 ± 4.9^a^	17.6 ± 8.9^b^	25.1 ± 8.9^c^	< 0.001

ANOVA indicates analysis of variance

^a, b, c^represented the same significance subset with post hoc comparisons

**Fig. 3:**
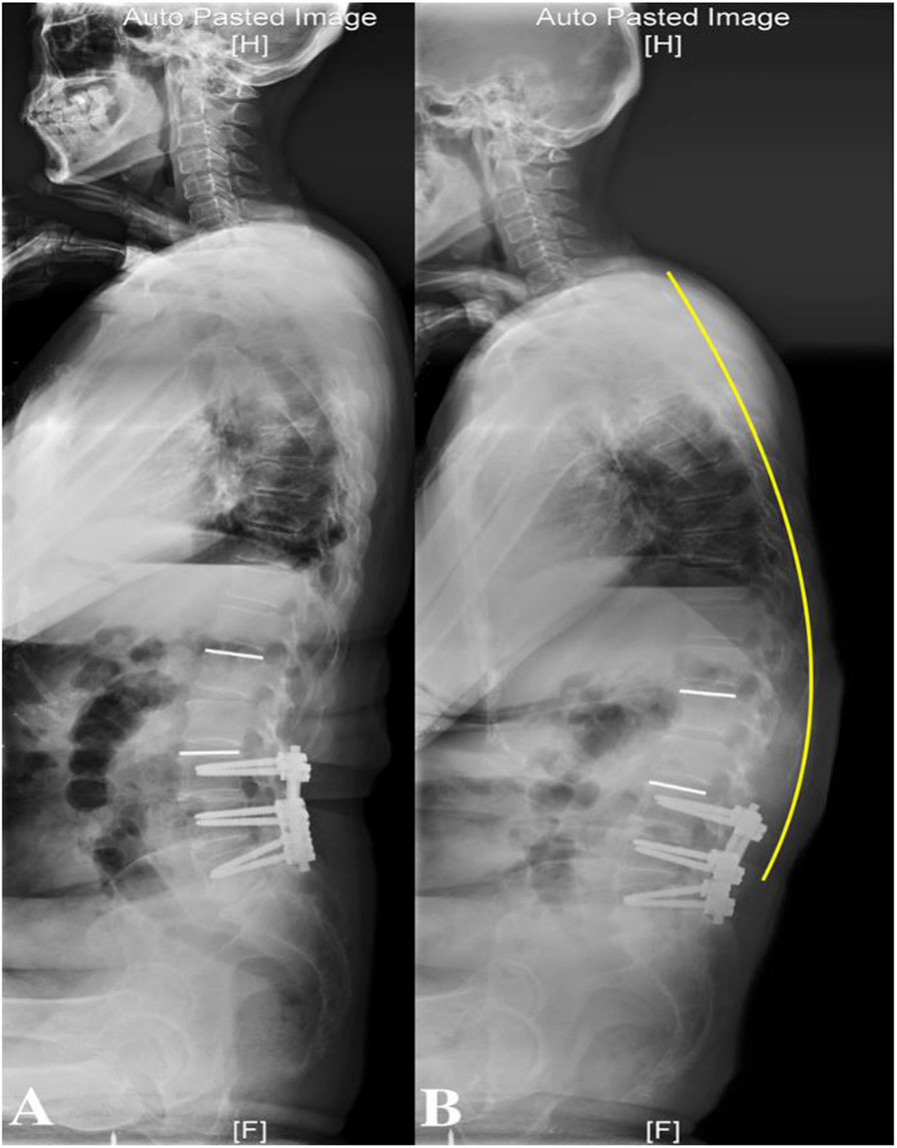
Female with 67 years old, 77 months follow-up after L3–5 fusion. The most noticeable changes of spinopelvic alignment occurred in the natural sitting position. A. URL was 12.2^°^ in erect sitting; B. URL was 5.3^°^ in natural sitting. The trunk slumped toward achieving a C-shaped sagittal profile (yellow line)

When changing from standing to sitting position at the final follow-up, all spinopelvic sagittal parameters with the exceptions of PI and TK were significantly altered. The most noticeable changes occurred in the natural sitting position; in this position, the spine slumped toward achieving a C-shaped sagittal profile for patients with both floating and lumbosacral fusions (Fig. 3). For patients with lumbosacral fusions, PT and TPA were significantly increased, while SS, LL, and URL were significantly decreased. For patients with floating fusions, in addition to the above parameters, LRL was also significantly decreased in the natural sitting position.

Physical parameters (sex, age, height, BMI, and PI) and surgical factors (lumbosacral fusion, fusion levels) associated with the changes in LL, unfused adjacent segment lordosis, and PT caused by standing to natural sitting alteration were determined using univariate analyses. As indicated in Table 2, ΔLL was significantly correlated with age, fusion levels, and FSL when changing from a standing to natural sitting position. ΔURL was significantly correlated with age and lumbosacral fusion. ΔPT was significantly correlated with PI and fusion levels. Multiple linear regression analysis demonstrated that only age and fusion levels reflected ∆LL, only age and lumbosacral fusion reflected ΔURL, and only PI reflected ΔPT. According to Table 3, with an increase in age and fusion levels, the ΔLL from a standing to natural sitting position was smaller. According to Table 4, with an increase in age, the ΔURL from a standing to natural sitting position was smaller. Lumbosacral fusion was found to play a positive role in the increase in ΔURL. According to Table 5, with an increase in PI, the ΔPT from a standing to natural sitting position was larger.

**Table 2:** Factors associated with the changes of LL, unfused residual lordosis and PT in relation to standing-to-natural sitting postural change

	ΔLL	ΔURL	ΔLRL	ΔPT
Sex	−0.012 (0.924)	0.011 (0.930)	−0.132 (0.537)	− 0.048 (0.708)
Age	−0.327 (0.009)	− 0.301 (0.017)	0.009 (0.968)	− 0.172 (0.176)
Height	0.107 (0.408)	0.041 (0.754)	0.191 (0.371)	0.043 (0.742)
BMI	0.190 (0.139)	0.085 (0.511)	0.037 (0.862)	0.103 (0.423)
Lumbosacral fusion	−0.078 (0.542)	0.300 (0.017)	–	0.001 (0.994)
Fusion levels	−0.393 (0.001)	−0.223 (0.079)	− 0.235 (0.268)	−0.311 (0.013)
FSL	−0.272 (0.031)	−0.157 (0.220)	0.234 (0.270)	−0.156 (0.223)
PI	0.148 (0.247)	0.101 (0.430)	−0.120 (0.577)	0.263 (0.038)

All values indicate the correlation coefficient (*p* value)

**Table 3:** The results of multiple linear regression analysis in influence factors of ΔLL in relation to standing-to-natural sitting postural change

	Regression coefficient	*P* value	Standardized coefficient
Intercept	42.62	< 0.001	0
Fusion levels	−5.32	0.005	− 0.34
Age	−0.28	0.017	−0.28

**Table 4:** The results of multiple linear regression analysis in influence factors of ΔURL in relation to standing-to-natural sitting postural change

	Regression coefficient	*P* value	Standardized coefficient
Intercept	25.70	< 0.001	0
Lumbosacral fusion	4.97	0.023	0.27
Age	− 0.29	0.004	− 0.35

**Table 5:** The results of multiple linear regression analysis in influence factors of ΔPT in relation to standing-to-natural sitting postural change

	Regression coefficient	*P* value	Standardized coefficient
Intercept	−6.56	0.250	0
PI	0.35	0.004	0.36

## Discussion

Previous studies have reported the differences between standing and sitting positions in asymptomatic subjects. The results indicated that in the sitting position, the curvature of LL decreased by 50% and PT increased by 25%, which can be summarized as a straightened curve in the lumbar region, pelvic retroversion, and forward displacement of sagittal balance [1–5, 11]. Hey et al. compared the spinal sagittal alignment in three weightbearing positions (standing, erect sitting, and natural sitting) in healthy subjects and found that LL decreased by approximately 80% in the natural sitting position; furthermore, the curvature of the trunk was vaguely C-shaped [3]. The current study also measured the spinopelvic sagittal alignment in three common weightbearing positions (standing, erect sitting, and natural sitting). We found that the sagittal alignment noticeably changed among positions, particularly with regard to LL and unfused adjacent segment lordosis. When changing to a natural sitting position, the entire thoracic region, unfused adjacent segments, and fusion segments were also vaguely C-shaped. Currently, almost all of the published studies on the target of alignment reconstruction and related mechanical complications were conducted in the standing position for reference [12–15]. But in daily life, patients’ physiological weight-bearing position is constantly changing between standing and sitting positions. The findings of this study in the natural sitting position could explain some of the problems observed after lumbar fusion surgeries. Lumbar fusion segments would prevent the spine into its natural shape in the natural sitting position. Instead, the curvature of trunk was vaguely C-shaped, the spine would inevitably be resulted in excessive stress in the adjacent segment area [16]. In natural sitting position, with decreased URL and LRL, the curvature of adjacent segments was naturally kyphotic. If combined with osteoporosis, this presumably could lead to complications such as implant loosening and proximal or distal junctional kyphosis and failure. These considerations were ignored in previous investigations of the etiological factors of junctional complications.

Spinal fusion renders the spine immobile in a fixed curvature; therefore, the spinal surgeon should pay close attention to the characteristics of changing alignment and the influencing factors in different weightbearing positions. Several studies have examined standing spinal alignment variability across age with the aim of discovering a more individually tailored strategy for sagittal realignment surgeries and the possible causes of junctional complications. In a previous study, for asymptomatic subjects, it was demonstrated that age significantly affected the change in LL when the position was changed from standing to sitting. With increased age, the reduction in LL was smaller [11, 17]. In this study, we also found that with increased age, ΔLL and ΔURL were both smaller after lumbar fusion. These findings indicated that the ability to compensate for the changes in lumbar alignment was reduced in elderly patients. We speculate that imbalance in sitting position can easily occur, indicating that the elderly patients would be unable to control the trunk when sitting down, and the trunk can easily tilt backward uncontrollably due to less ability to compensate alignment in the adjacent segments. To achieve balance, the demand on the paraspinal muscles to maintain spinal alignment naturally increases, and it becomes reasonable to assume that these elderly patients will have greater back pain or fatigue in the sitting position. Proposed age-adjusted and sitting values for sagittal realignment surgeries may further influence future surgical strategies [18–20].

Maekawa et al. performed an imaging analysis of sagittal alignment in the standing and sitting positions in 253 subjects with an average age of 53.6 years [21]. The results revealed that when changing to a sitting position, the changes in sagittal lumbo-pelvic alignment (ΔPT and ΔLL) were found to be regulated by the degree of PI. An asymptomatic subject with low PI in the standing position would be less capable of performing changes in alignment between the sitting and standing positions. However, in the current study, with the exception of ΔPT, the changes in LL and unfused adjacent segment lordosis were not found to be regulated by PI. We speculate that the fused segments resulted in the loss of regulation between PI and ΔLL. Although PI is an anatomical parameter and plays an important role in sagittal realignment, age is a more important influencing factor than PI when we consider the changes in LL and unfused adjacent segment lordosis in the sitting position.

The current study had some limitations. First, only patients who had undergone short lumbar fusions were included. Thus, the features of sagittal alignment in the sitting position after long fusions will be discussed in future studies, particularly in the natural sitting position. Secondly, as we only investigated imaging changes without clinical scores and a biomechanical basis in this study, we cannot simply apply the conclusions drawn from these changes to junctional complications. This will be studied in the future. These previously ignored considerations may provide a new theoretical basis for the surgical strategy and the etiology of junction complications.

## Conclusions

We studied the changes in spinopelvic sagittal alignment in different positions (standing, erect sitting, and natural sitting) after posterior lumbar fusion. The most noticeable changes occurred in the natural sitting position, with the spine slumped toward achieving a C-shaped sagittal profile. Analysis revealed that age, fusion levels, and lumbosacral fusion significantly influenced the differences in LL and URL between standing and natural sitting positions. These characteristics of spinopelvic alignment in the natural sitting position should be fully considered in the surgical reconstruction of sagittal alignment and the identification of etiological factors of junctional complications.

## Data Availability

The datasets will be available from the corresponding author if required.

## Abbreviations

PLIF: Posterior lumbar interbody fusion
BMI: Body mass index
PI: Pelvic incidence
PT: Pelvic tilt
SS: Sacral slope
LL: Lumbar lordosis
FSL: Fusion segment lordosis
URL: Upper residual lordosis
LRL: Lower residual lordosis
TK: Thoracic kyphosis
T1PA: T1 pelvic angle
